# A Rare Case of Spontaneous Intracranial Hypotension Secondary to C4-C5 and T12 Dural Tears Following a Yoga Class

**DOI:** 10.7759/cureus.71016

**Published:** 2024-10-07

**Authors:** Antony Antypas, James Baker, Andrew Koo

**Affiliations:** 1 Radiology, Leeds Teaching Hospitals NHS Trust, Leeds, GBR; 2 Emergency Medicine, Bankstown-Lidcombe Hospital, Sydney, AUS; 3 Radiology, Harrogate and District NHS Foundation Trust, Harrogate, GBR

**Keywords:** accidental dural tear, constructive interference in steady-state (ciss), emergency med, general radiology, intracranial hypotension, neuroradiology, neurosurgery, sports injury rehabilitation, yoga therapy

## Abstract

Spontaneous intracranial hypotension (SIH) is a potentially debilitating condition caused by cerebrospinal fluid (CSF) leakage through dural tears, leaking meningeal diverticula, or CSF-venous fistulae. This case report describes two yoga-induced dural tears leading to spontaneous SIH. Diagnosing SIH due to dural tears and CSF leaks can be challenging, but magnetic resonance imaging (MRI) aids in confirmation. A woman in her 30s presented to the Emergency Department with a three-week history of sudden, right-sided orthostatic headache following a yoga session, accompanied by tinnitus, visual disturbances, and vomiting. A head and C-spine MRI, including a high-resolution constructive interference in steady state sequence, revealed pachymeningeal enhancement and dural tear at the C4/C5 interspinous process region. Despite conservative management and epidural blood patches, surgical intervention was required to treat the CSF leaks. This report underscores the importance of clinician awareness regarding yoga-associated SIH. Prompt diagnosis and accurate radiological assessment are crucial, and educating patients about the risks during yoga can aid in early detection and treatment.

## Introduction

Spontaneous intracranial hypotension (SIH) remains a poorly understood condition, with misdiagnoses being common. SIH is a highly disabling syndrome secondary to cerebrospinal fluid (CSF) leak [1]. This may be caused by a dural tear, leaking meningeal diverticulum, or CSF-venous fistula [2]. The incidence of SIH has been reported as being between 1 in 50,000 and 5 in 100,000 people [3]. SIH should be considered in any patient presenting with an orthostatic ‘end-of-day’ headache with improvement when lying flat [4]. SIH is diagnosed when a headache has developed spontaneously and in temporal relation to a CSF leak (evident on imaging) and/or CSF hypotension (lumbar puncture opening pressure <60 mm) [5]. Early recognition of SIH is vital, utilising radiological investigations, including computed tomography (CT) myelography or magnetic resonance imaging (MRI). Conservative management is suggested in the first instance with analgesia, intravenous fluids, caffeine, or epidural blood patches (EBPs) offered to those with debilitating symptoms. Neurosurgical management is reserved for those who fail first-line active treatment. This case report describes the first case of a Yoga-associated SIH caused by two areas of CSF leak.

## Case presentation

A 38-year-old woman attended the emergency department with a three-week history of sudden, right-sided headache and neck pain directly following an intense yoga class. The pain had worsened despite various prescribed analgesic medications from general practitioners before presentations. It was postural, aggravated by standing or leaning forward, and relieved when lying flat. Standing for 20 minutes resulted in dizziness and light-headedness, accompanied by right-sided tinnitus and blurred vision. The patient also experienced intermittent photophobia, phonophobia, and cognitive cloudiness which is sometimes referred to as ‘brain fog’. The patient had no significant medical or family history.

**Figure 1:**
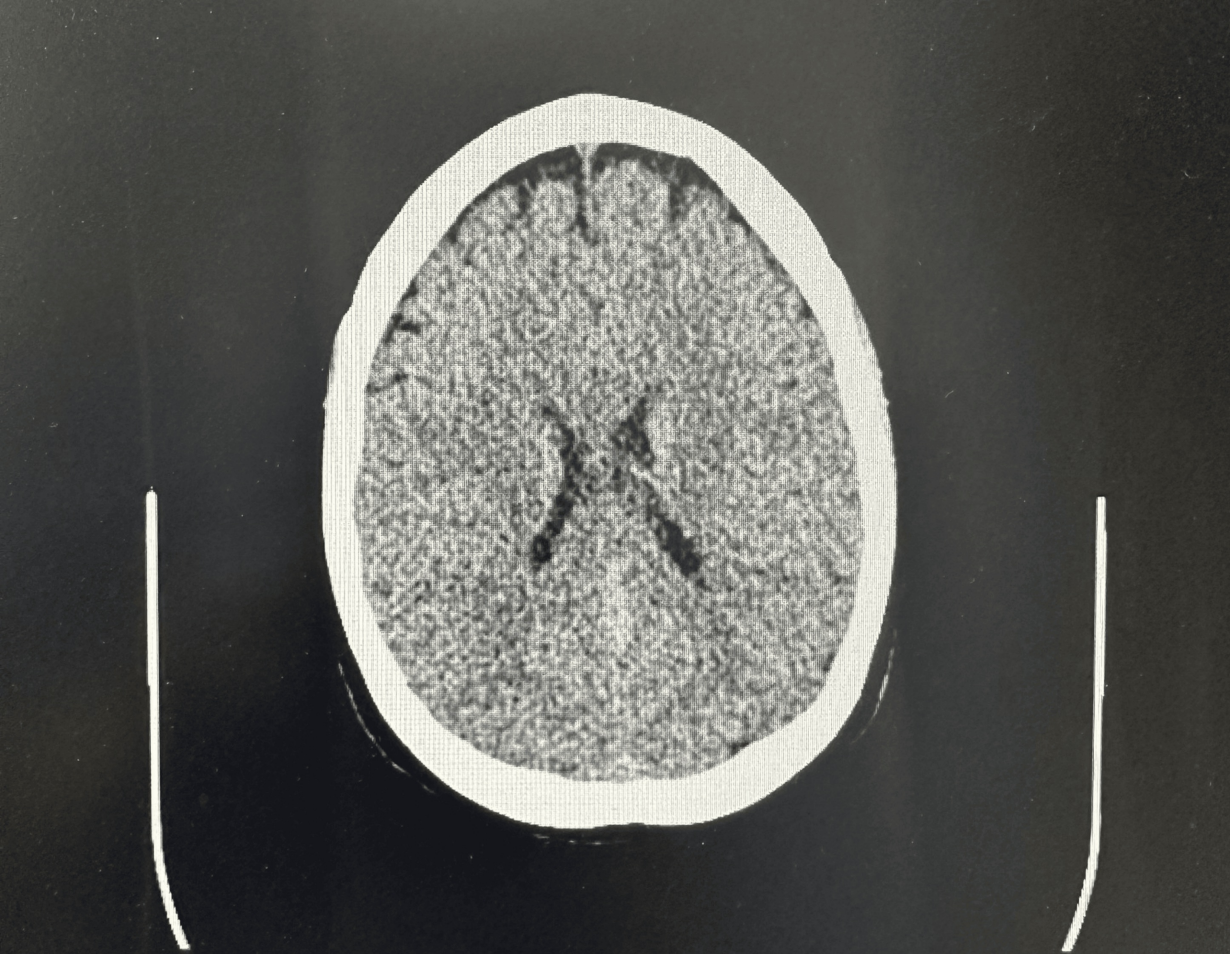
CT axial section showing a normal scan.

**Figure 2:**
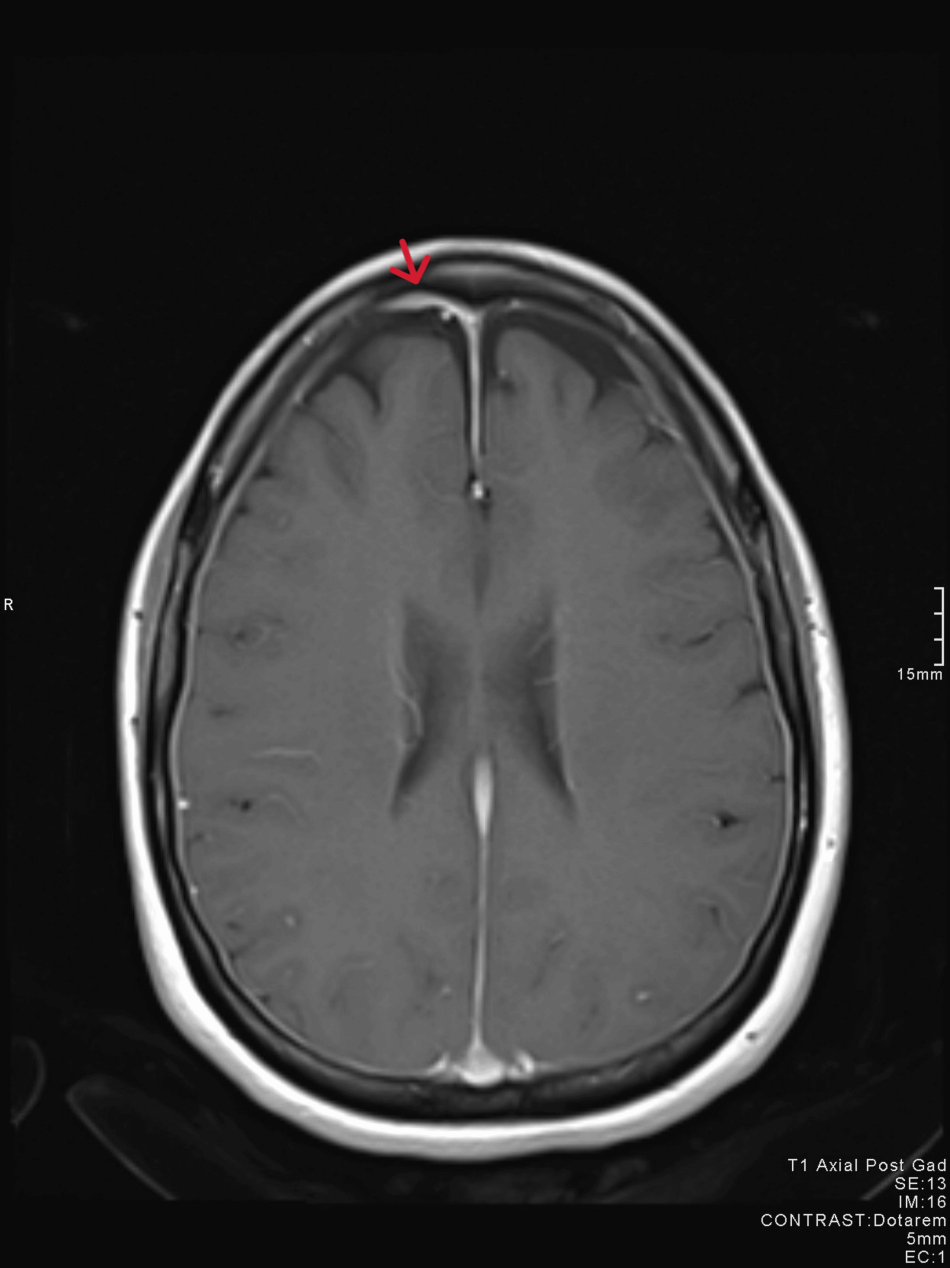
MRI axial view showing pachymeningeal enhancement.

**Figure 3:**
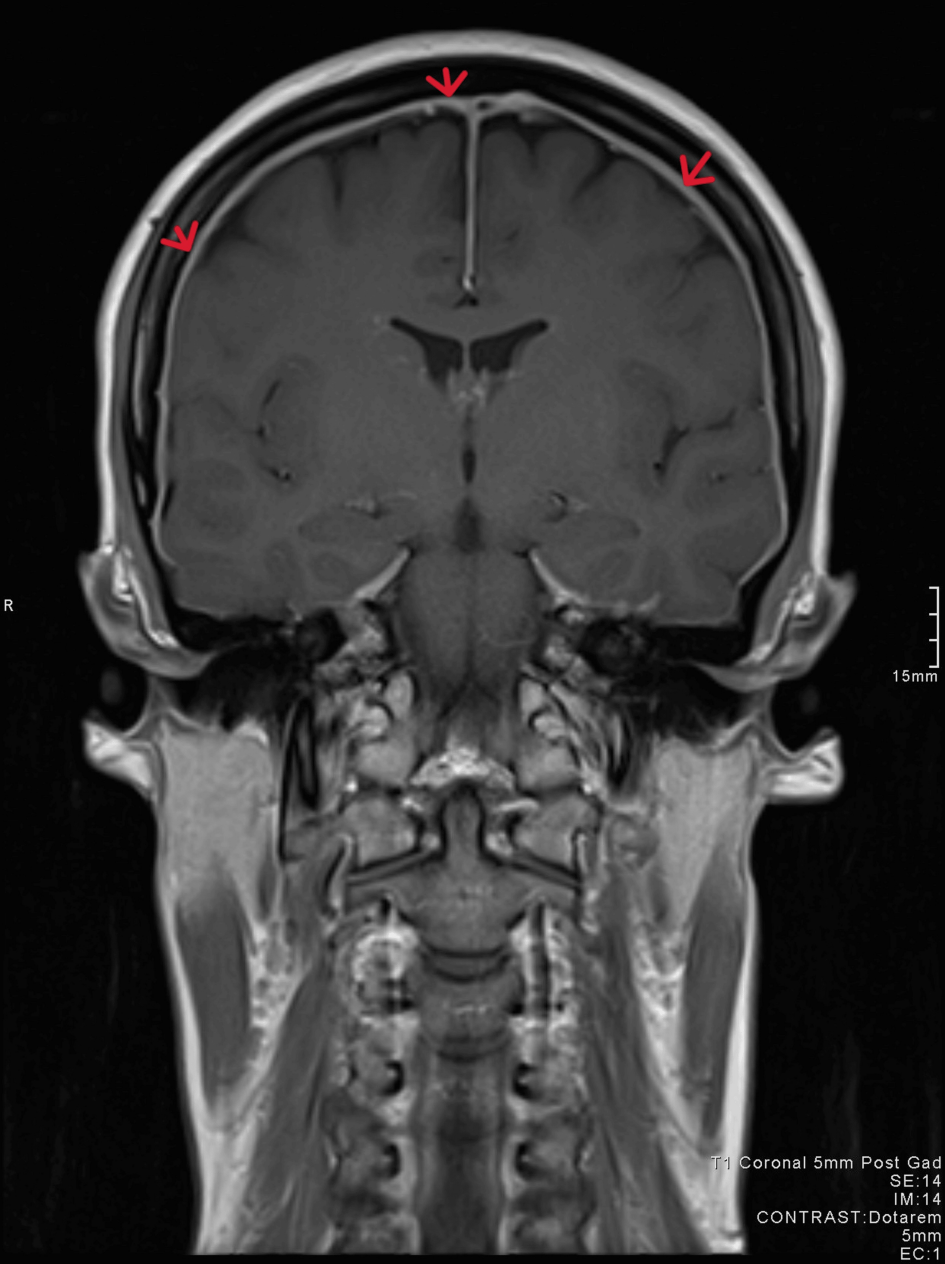
MRI coronal view showing pachymeningeal enhancement.

**Figure 4:**
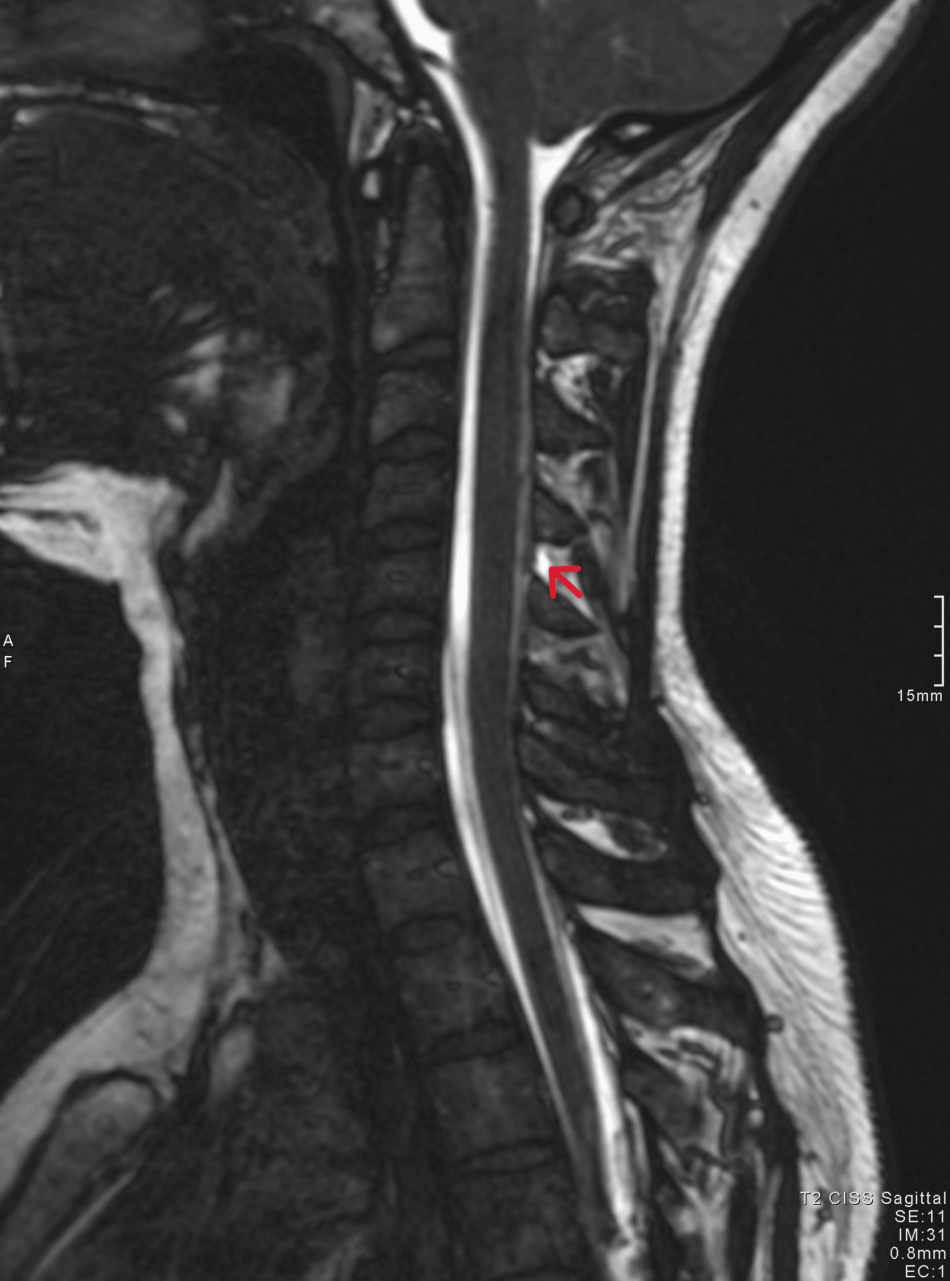
MRI constructive interference in steady-state sequence sagittal view showing fluid signal at the level of the C4/C5 interspinous process.

**Figure 5:**
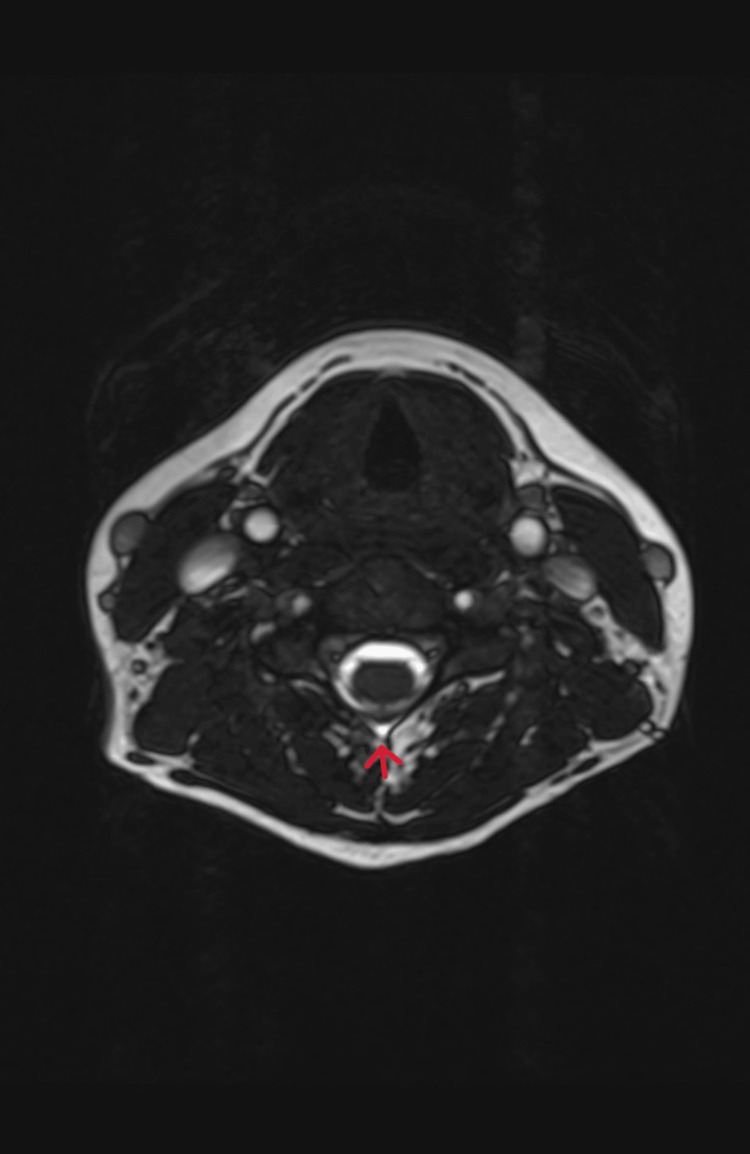
MRI constructive interference in steady-state sequence axial view showing fluid signal at the level of the C4/C5 interspinous process.

The patient’s blood results and observations were normal. Neurological examination and fundoscopy showed no abnormalities. Following an assessment of her symptoms, she underwent a CT scan of the head which showed no abnormality (Figure 1). The following day, further investigation was performed with an MRI of the head and C-spine, complemented by an additional constructive interference in steady-state (CISS) sequence. The MRI of the head revealed pachymeningeal enhancement with thickening (Figures 2, 3). Additionally, a 6 mm focal extradural fluid signal was noted posteriorly on the CISS sequence, suggesting a dorsal site of dural tear at the C4/C5 interspinous process region (Figures 4, 5).

The patient was referred to the acute medical clinic at the local tertiary centre for diagnosis confirmation and management. She underwent conservative treatment, including regular doses of caffeine (50-100 mg six-hourly) and Naproxen (250 mg eight-hourly), complimented with rest and hydration. She was monitored by the community assessment team and seen in the acute medical clinic regularly. The anaesthetics team considered an EBP, despite its unconventional use at the cervical spine level. The patient underwent this procedure and initially noted significant improvement in her symptoms. She was able to mobilise for up to two hours, a significant improvement from her pre-procedure mobility. Unfortunately, the symptoms re-emerged, prompting a second, yet unsuccessful attempt at the blood patch. A referral to the neurosurgical department within the tertiary centre was made. A subsequent CT myelogram scan was organised to evaluate the persistent, refractory symptoms which revealed an additional dural tear at the level of T12, as well as signs of improvement of the previous tear at C4/C5. She underwent a repair of the dural tear at the level of T12 traversing the site of a T12 hemilaminectomy using a direct suture technique. The patient was given postoperative advice of rest, plenty of hydration, and analgesics, and was informed that recovery may take 6-12 months. She was reviewed at regular intervals and found to be recovering, gradually, to her usual level of activity, two years after she first sustained her dural tears.

## Discussion

SIH, characterised by its complex symptoms, is a poorly understood medical condition. Its frequent underdiagnosis can potentially lead to life-threatening complications if missed [6]. Predominantly, it stems from a tear in the dural sac, leading to a CSF leak, either spontaneous or caused by trauma. Several risk factors can precipitate this condition, including trauma to the spine from events such as head and spinal surgeries, lumbar punctures, and meningeal infections. Additionally, certain physical activities such as yoga, Pilates, and chiropractic manipulation have been recognised as potential triggers [7-9]. It is hypothesised that excessive stretching in yoga may elevate the risk of dural tears by imposing additional strain on the spinal dura mater, which is particularly exacerbated among individuals with pre-existing spinal pathology or a history of spinal surgery. Additionally, underlying connective tissue disorders affecting fibrillin or elastin, such as Marfan syndrome, may predispose individuals to dural weakness [1,10,11].

The cardinal symptoms of SIH consist of orthostatic headaches, worsened within 15-30 minutes of standing or sitting upright, and may be associated with cervical neck pain, nausea, vomiting, photophobia and phonophobia [6,12]. Despite these symptoms, a dural tear may remain undiagnosed unless manifesting with symptoms of intracranial hypotension [12]. Radiological imaging such as CT and MRI scans can aid in diagnosing SIH and dural leak by providing confirming evidence. The MRI radiological findings of this condition are characterised by the thickening of the pachymeningeal layer, increased venous blood volume in the dural venous sinuses, subdural effusions and haematomas, and widespread cerebral oedema [12-14]. With the progression of the disease, the thickening of the pachymeningeal layer may become less apparent on scans, therefore lack of enhancement should not exclude SIH diagnosis [14,15]. Another indicator of SIH is the enlargement of the pituitary gland. When there is a tear in the dura, CSF leaks out, resulting in visible signs of reduced CSF volume, such as sagging brainstem, drooping of the splenium of the corpus callosum, and decreased fluid within the optic nerve sheath [4,12,14,16].

When a dural tear is suspected, it is necessary to undergo a fat-saturated T2 MRI of the spine to provide a clear picture of the accumulation of CSF in the epidural space. Although T1 imaging can also show a reduced signal in the epidural space, it may be more difficult to detect [17]. Additional CISS sequence MRI offers excellent spatial resolution, enabling the identification of dural tears through the high contrast between CSF and soft tissues, using the CISS imaging technique can help identify the sites of dural tears with high sensitivity and specificity of 89% and 95%, respectively [18]. Alternatively, CT myelography has been found to be a very useful imaging modality to identify the location of CSF leak in suspected SIH. The contrast material enters the thecal sac providing a means to identify the specific site of CSF leak into the epidural space, this imaging technique is commonly used before surgical intervention, if conservative measures have failed [17].

Initial management embraces conservative approaches, including rest, increased caffeine consumption, and analgesics. Guiding patients to avoid triggers such as sitting upright may alleviate symptoms. If symptoms do not improve, an epidural blood patch is indicated [19,20]. Epidural blood patches are usually used in the lumbar region but can be performed higher up the spine under careful and specific circumstances with good effect to seal off the site of the leak [19,20]. Neurosurgical intervention is used as a last resort, requiring radiological identification of the exact site of active CSF leaks [3].

## Conclusions

SIH is a commonly overlooked cause of headaches. Early recognition and appropriate radiological investigations are crucial for timely diagnosis and treatment. Misdiagnosis can lead to progressive and debilitating symptoms. Clinicians should educate patients, particularly those engaged in low-risk activities such as Yoga, about the signs and risks of SIH and CSF leaks. Radiologists play a key role in identifying SIH and dural tears using the correct imaging modality and sequence while considering all relevant radiological signs. Increased awareness among both clinicians and patients is crucial to avoid unnecessary tests and treatment delays.
